# Computational Assay of H7N9 Influenza Neuraminidase Reveals R292K Mutation Reduces Drug Binding Affinity

**DOI:** 10.1038/srep03561

**Published:** 2013-12-20

**Authors:** Christopher J. Woods, Maturos Malaisree, Ben Long, Simon McIntosh-Smith, Adrian J. Mulholland

**Affiliations:** 1 Centre for Computational Chemistry, School of Chemistry, University of Bristol, Bristol, UK; 2 Department of Computer Science, University of Bristol, Bristol, UK

## Abstract

The emergence of a novel H7N9 avian influenza that infects humans is a serious cause for concern. Of the genome sequences of H7N9 neuraminidase available, one contains a substitution of arginine to lysine at position 292, suggesting a potential for reduced drug binding efficacy. We have performed molecular dynamics simulations of oseltamivir, zanamivir and peramivir bound to H7N9, H7N9-R292K, and a structurally related H11N9 neuraminidase. They show that H7N9 neuraminidase is structurally homologous to H11N9, binding the drugs in identical modes. The simulations reveal that the R292K mutation disrupts drug binding in H7N9 in a comparable manner to that observed experimentally for H11N9-R292K. Absolute binding free energy calculations with the WaterSwap method confirm a reduction in binding affinity. This indicates that the efficacy of antiviral drugs against H7N9-R292K will be reduced. Simulations can assist in predicting disruption of binding caused by mutations in neuraminidase, thereby providing a computational ‘assay.'

On the 31^st^ March 2013 Chinese authorities reported the first three cases of a novel influenza virus of subtype H7N9^1^,^2^. According to the World Health Organisation (WHO), as of 12^th^ August 2013, patients infected with this strain have been confirmed in China and Taiwan, with 135 cases and 44 fatalities. H7N9 is an avian influenza virus transmissible from poultry to humans^3^,^4^, and while there are only signs of human-to-human transmission^5^, its emergence is a serious cause for concern^6^. H7N9 is the first N9-type influenza to infect humans. This suggests that the general population will have little natural immunity to the virus, giving the potential for any outbreak to turn into a serious influenza pandemic^7^. To help understand the virus, detailed patient and viral genome sequence data from those infected has been made available rapidly and publicly^1^,^8^. The timely release of this data has allowed copious studies of the virus to be performed^9^,^10^,^11^,^12^, providing scientists and health professionals with an early and detailed picture of this emerging threat. Here we report the first structural study into the structure and dynamics of a target protein of H7N9 influenza, neuraminidase. Since 2010, inhibitors of neuraminidase are the only class of antivirals recommended by the WHO for prophylaxis of influenza A and B infections^13^. Understanding the structure of neuraminidase in H7N9 influenza, and its interactions with available drugs is required to monitor the emergence of drug-resistant strains, and to inform the development of new treatments.

The main drugs used for antiviral treatment of H7N9 are the neuraminidase inhibitors oseltamivir (Tamiflu®), zanamivir (Relenza®) and peramivir^14^. These drugs provide the first line of defence against H7N9, until a vaccine can be developed^9^. The structures of these drugs, together with the location of important functional group substituents are shown in figure S1 in supporting information. Oseltamivir and zanamivir have been widely deployed in past influenza outbreaks^15^, with large stockpiles built up by several governments^16^,^17^. Oseltamivir is taken orally, zanamivir via a nasal spray, while peramivir is newly licensed in only a small number of countries, and must be taken via injection^13^. Initial laboratory tests of drug binding, and successful treatment of H7N9 patients with oseltamivir in the field, suggest that the drugs are effective against H7N9 neuraminidase. However, neuraminidase is known to be capable of mutating to a multitude of drug-resistant forms^13^,^18^,^19^. It is important to be able to predict whether any of the changes in sequence observed between the samples of H7N9 collected lead to structural changes in neuraminidase that could disrupt drug binding, and therefore provide early markers of emerging drug resistance. Here we show how computational assays can be used to predict and rationalize how differences in the collected sequences of H7N9 affect drug binding at the molecular level. This demonstrates how computational assays can complement the collection and publication of neuraminidase sequences and other experimental and clinical work, by providing a rapid screen to test for disruption of drug binding mode and reduction of drug binding affinity. The results also provide insight into how changes in sequence disrupt drug binding, thereby providing structural information that will be useful in the design of the next generation of neuraminidase inhibitors.

Complete viral genomes of samples of H7N9 from patients, birds and the environment have been sequenced and made publicly available via the Global Initiative on Sharing All Influenza Data (GISAID EpiFlu™) database (www.gisaid.org). The four sequences available at the start of this study (A/Shanghai/1/2013, A/Shanghai/2/2013, A/Anhui/1/2013 and A/Hangzhou/1/2013) contain neuraminidases that show high sequence similarity to many N9 type strains. Analysis showed high similarity to H11N9 neuraminidase (A/Tern/Australia/G70C/1975), with the sequences differing by only 20 amino acid residues in the ‘head' of the protein (residues 83–468,18 95% sequence identity). H11N9 neuraminidase has been well studied, with crystallographic structural data available of it bound with all of the available neuraminidase antivirals. Figure 1 shows the approximate spatial location of key active site residues in the head region of neuraminidase in relation to the main functional groups of the antiviral drugs. All of the four sequences of H7N9 are identical in the head region, with the exception of A/Shanghai/1/2013, which differs only by a single substitution of arginine to lysine at position 292 (H7N9-R292K, using N2 numbering: see table S1 in supporting information). All of the differences in sequence between H7N9 and H11N9 neuraminidase are far from the enzyme active site (figure 2). However, the arginine to lysine change in H7N9-R292K is in the active site, and involves a residue that is known to form a salt bridge with the carboxylate group of each of the antivirals (figure 2). The R292K mutation is known to confer drug-resistance in many strains of influenza^20^,^21^,^22^. Studies of N9 neuraminidase from H11N9 influenza (in a reassortant H1N1 virus, NWS/G70C) show that the R292K mutation confers drug resistance. Experimental binding assays of this neuraminidase reveal that the mutation reduces the binding affinity of oseltamivir and peramivir by several orders of magnitude, but only has a small effect on the binding of zanamivir^20^,^21^,^22^. The experimental ratio of the mutant to wildtype IC50s of the drugs are: oseltamivir 6,500^20^, zanamivir 55^20^ and peramivir 1,000^21^. As the R292K mutation reduces binding affinity in H11N9 neuraminidase, it is important to investigate whether R292K affects drug binding to H7N9. While the presence of the R292K substitution indicates the potential for the H7N9-R292K strain to show disrupted drug binding, it is not possible from sequence data alone to give a definitive prediction. Certainly, knowledge of the sequence alone does not provide sufficient information to predict the magnitude of any reduction in binding affinity. To answer these questions, we have performed computational assays involving multiple 70 nanosecond (ns) molecular dynamics simulations of oseltamivir, zanamivir and peramivir bound to H11N9, H7N9 and H7N9-R292K neuraminidase, together with absolute binding free energy calculations using the WaterSwap method^23^. WaterSwap calculates condensed phase absolute protein-ligand binding free energies using a dual topology reaction coordinate that swaps the ligand with an equivalent volume of water in the protein binding site. The advantage of WaterSwap is that it allows calculation of absolute binding free energies. This simplifies investigation of the affect of protein mutation on binding compared to relative binding free energy methods, as differences in absolute binding free energies capture changes in binding mode of the ligand upon protein mutation. This assay required approximately two weeks of computation, using readily accessible compute resources. The results indicate that molecular dynamics simulations coupled with binding affinity calculations with WaterSwap can provide a rapid and cheap predictive assay to detect disrupted drug binding in neuraminidase in emerging influenza strains.

## Results

H11N9 neuraminidase has been studied extensively, with X-ray structures available of it bound to oseltamivir (PDB code 2QWK^20^), zanamivir (1NNC^24^) and peramivir (1L7F^21^). The high sequence identity (95%) of H7N9 to H11N9 allowed us to use in-silico point mutations from these crystallographic structures to construct structural models of each of the drugs bound to both H7N9 and H7N9-R292K. A computational assay involving nine molecular dynamics simulations was performed using these models: oseltamivir, zanamivir and peramivir bound to H11N9, H7N9 and H7N9-R292K neuraminidase. The simulations were run with explicit solvent. Using graphics processors (GPUs), it was possible to simulate 70 nanoseconds of dynamics of each system within one week (three simulations per neuraminidase strain, 630 ns in total in seven days). WaterSwap^23^ absolute binding free energy calculations of the three drugs bound to H7N9 and H7N9-R292K followed these, using starting structures taken every 10 ns from 20–70 ns from the dynamics simulations. These calculations were completed in one week, on a small compute cluster (36 binding calculations using 36 compute nodes). This shows that useful results can be delivered on the timescale of a few days, using relatively modest compute resources. The simulations provide structural understanding of the molecular interactions between the proteins and the drugs, enabling analysis of the effects of mutation on protein dynamics and drug binding^25^.

Structural analysis of the effect of point mutation on the structure of neuraminidase was performed using calculation of root mean square fluctuations (RMSF) and deviations (RMSD) (see figure S2–S5 in supporting information), and visual inspection of the protein backbone (figures S3–S5). Analysis of RMSF show that the point-mutated residues show similar fluctuations in the H7N9 and H7N9-R292K trajectories to those seen in the H11N9 trajectories. There are no significant peaks, indicating that the point-mutated residues are fluctuating around a stable conformation, and are not triggering any large structural changes in the backbone of the proteins. Combined with analysis of RMSD and visualization of the protein backbone, this shows that there is little difference in protein dynamics and global conformation between H7N9, H7N9-R292K and H11N9. The structure of H7N9 and H7N9-R292K neuraminidase is revealed to be homologous to that for H11N9, with the differences in sequence having negligible effect. While the protein backbone structure of H7N9 and H7N9-R292K shows high similarity to H11N9, the change from arginine to lysine in H7N9-R292K significantly disrupts the binding mode of the three drugs. This disruption is structurally similar to the disruption observed experimentally for the binding of the drugs to H11N9-R292K^20^,^21^, and is discussed in detail in the following sections.

### Oseltamivir complexes

**Figure 3:**
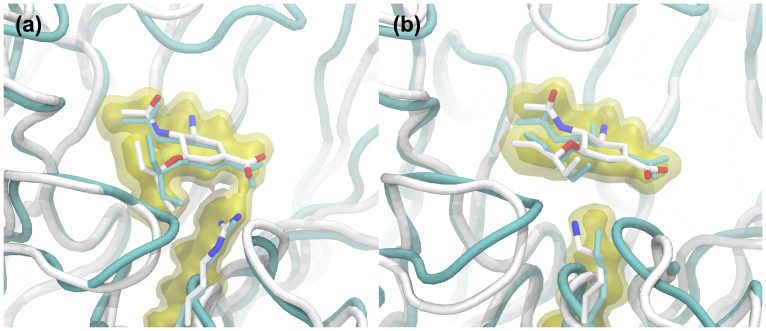
Structure of oseltamivir bound to (a) H7N9 and (b) H7N9-R292K neuraminidase. In cyan is shown the experimental X-ray structure of oseltamivir bound to (a) H11N9 (2QWK^20^) and (b) H11N9-R292K (2QWE^20^), while in white is shown a representative snapshot taken from simulation. The average location of oseltamivir and arginine/lysine during the simulation is shown using isosurfaces, with the volume of space occupied on average during 90% (solid yellow) and 60% (transparent yellow) of the trajectory displayed. This shows that lysine in the H7N9-R292K simulation is located to disrupt binding of the bulky group of oseltamivir, with the bulky group twisted up, out of the bulky group binding pocket. This is in agreement with the X-ray structure (2QWE) of H11N9-R292K.

Visualisation and analysis of the simulations shows that the twenty point mutations necessary to create the H7N9 model have a negligible effect on the structure and dynamics of neuraminidase, with H7N9 revealed to be structurally homologous to H11N9. Visualization of oseltamivir in the active site reveals the same picture. The oseltamivir-bound simulations show high similarity between the binding modes of the drug to H7N9 and H11N9 neuraminidase (figure 3a). There is excellent agreement between the two simulations, and also with the crystallographic structure. This helps validate the simulation and provides confidence in the conclusion that H7N9 and H11N9 neuraminidase are structurally similar. While there is excellent agreement between the binding mode of oseltamivir in the H7N9 and H11N9 simulations, a different picture is revealed for H7N9-R292K. The change from arginine to lysine significantly disrupts binding of oseltamivir. Instead of forming a salt bridge to the carboxylate group, as observed in the H11N9 X-ray crystal structure, and in the H11N9 and H7N9 simulations (figure 3a), lysine moves to disrupt binding of the hydrophobic bulky group of the drug (figure 3b). The bulky group rotates up and moves out of the hydrophobic pocket. This is in strong agreement with the X-ray structure of oseltamivir bound to the R292K mutant of H11N9, which is shown in figure 3b for comparison (PDB code 2QWE^20^). The rotation of the bulky group can be monitored by measuring the angle between it and the plane of the central ring of oseltamivir. In the case of H11N9 and H7N9, during simulation, the bulky group points downwards, fluctuating between angles of −30° to −90° relative to the ring (figure S6 in supporting information). In contrast, in H7N9-R292K, the bulky group points upwards, fluctuating between angles of 10° to 40°, in agreement with the angle of 20° observed in the H11N9-R292K X-ray structure (figure S6). Disruption of drug binding is confirmed by the WaterSwap calculations^23^, which show that the R292K mutation reduces the binding affinity of oseltamivir to neuraminidase by 3.0 ± 1.2 kcal mol^−1^ (a table of all of the binding free energies is provided in table S2).

### Zanamivir complexes

**Figure 4:**
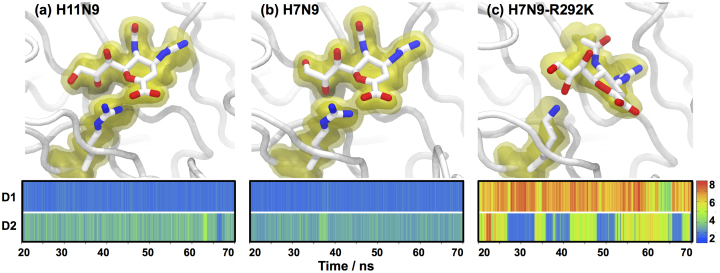
Simulation structures of zanamivir bound to (a) H11N9, (b) H7N9 and (c) H7N9-R292K neuraminidase. All figures are rendered from the same viewpoint, showing that the lysine residue in H7N9-R292K hydrogen bonds with the bulky group of zanamivir, shifting the drug up and to the right compared to H11N9 and H7N9. The figures show representative snapshot structures from the simulations, with the average location of zanamivir and arginine/lysine shown using isosurfaces, with the volume of space occupied during 90% (solid yellow) and 60% (transparent yellow) displayed. The graphs show the distance between the arginine/lysine and the carboxylate group (D1) and bulky group (D2) on zanamivir. Distances are shown on a color scale from 2–8 Å, with blue-green values indicating the presence of a hydrogen bond or salt bridge. These show that while arginine interacts directly with both the carboxylate and bulky groups of zanamivir, lysine hydrogen bonds only sporadically with the bulky group.

As seen for oseltamivir, the zanamivir-bound simulations confirm that H7N9 and H11N9 neuraminidase are structurally homologous, with little difference seen between the binding modes of zanamivir to the two proteins (figures 4a and 4b). In addition, the simulation of zanamivir bound to H11N9 neuraminidase reproduces the crystallographic structure well, giving confidence in the computational model. The H7N9-R292K simulation shows that the change from arginine to lysine significantly disrupts binding of the drug. In the H11N9 and H7N9 neuraminidases, arginine interacts directly with both the carboxylate group and the hydrophilic bulky group of zanamivir. In contrast, the smaller size of lysine compared to arginine means that it can only interact directly with one of these groups. In the H7N9-R292K simulation, lysine292 moves from the carboxylate group to form a hydrogen bond with the bulky group of zanamivir (figure 4c). This is seen in the distance plots in figure 4, which show the distance between the heavy atoms of the arginine/lysine and the carboxylate and bulky groups of zanamivir in simulations of the three enzymes. Hydrogen bonding of lysine to only the bulky group causes a change in binding mode of zanamivir in H7N9-R292K neuraminidase. This is shown clearly by comparing figures 4a–4c, which are visualized from the same viewpoint. This change in binding mode agrees with experimental structural studies of H11N9-R292K, in which lysine292 is seen to hydrogen bond with the bulky group, and zanamivir shifts binding mode^20^. WaterSwap calculations predict that this change in binding mode is accompanied by a reduction in binding affinity of 4.4 ± 3.1 kcal mol^−1^ for zanamivir to H7N9-R292K compared to H7N9 neuraminidase.

### Peramivir complexes

**Figure 5:**
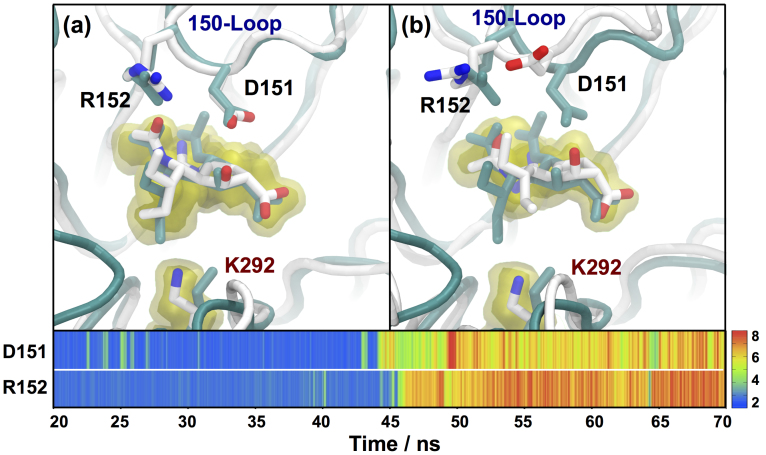
Structures of peramivir bound to H7N9-R292K neuraminidase, showing representative snapshots and the average structure between (a) 20–40 ns and (b) 40–70 ns. The average location of peramivir and lysine is shown using isosurfaces, with the volume of space occupied during 90% (solid yellow) and 60% (transparent yellow) of the two time periods of the simulation displayed. The H11N9-R292K X-ray structure (1L7H^21^) is shown in cyan. This shows that the simulation closely matched the X-ray structural data in the first part of the simulation. After 40 ns, the 150-loop partially opened, leading to a loss of interaction between peramivir and D151/R152, and a change in binding mode of the drug. The shortest distance between the hydrogen bond donors and acceptors of peramivir (guanidinium and hydroxyl groups) to the D151 and R152 residues on the 150-loop are shown on a color-scale from 2–8 Å, with blue-green values indicating the presence of a salt bridge or hydrogen bond. This shows that the interactions between peramivir and the two 150-loop residues were lost at approximately 45 ns. Equivalent figures for peramivir bound to H11N9 and H7N9 neuraminidase are given in figure S8 in supporting information.

The peramivir-bound simulations show that the binding mode of the drug to H7N9 and H11N9 neuraminidase is similar, reinforcing the conclusion that H7N9 is homologous with H11N9 (figure S7 in supporting information). However, as was observed for oseltamivir and zanamivir, the change from arginine to lysine in H7N9-R292K neuraminidase significantly disrupts binding. The peramivir-bound H7N9-R292K simulation shows that lysine292 does not interact with the carboxylate group of peramivir, but instead moves towards the bulky group. This movement of lysine from interacting with the carboxylate group to interacting with the bulky group of the drug is seen in all of the H7N9-R292K simulations, and all of the H11N9-R292K crystallographic structures. Zanamivir has a hydrophilic bulky group, to which lysine is able to form favourable hydrogen bonding interactions. Peramivir, like oseltamivir, has a hydrophobic bulky group. For oseltamivir, interaction with lysine caused the bulky group to be rotated out of the pocket. For peramivir, the bulky group remained in the pocket, in agreement with the X-ray structure (1L7H^21^) of peramivir bound to H11N9-R292K (figure 5). This structure was maintained for the first 20 ns of the simulation, until the direct interactions between peramivir and the D151 and R152 residues on the 150-loop of neuraminidase were broken. Partial opening of the 150-loop, and a change of binding mode of peramivir away from the crystallographic structure followed. The 150-loop is a structurally important part of neuraminidase, and is known to be capable of opening and closing in group 1 neuraminidases^25^,^26^,^27^ While it is less flexible in group 2 neuraminidases (N2, N3, N6, N7 and N9), combined experimental/computational studies now suggest that it does move between a closed and partially opened state^28^. In previous work, we demonstrated that opening of the 150-loop can be seen in timescales comparable to these simulations, and was indicative of reduced binding affinity caused by a drug resistant mutation^25^. A change of binding mode and partial opening of the 150-loop was not observed in the simulations of peramivir binding to H11N9 or H7N9 neuraminidase. WaterSwap^23^ binding calculations indicate that the change in binding mode reduces the binding free energy of the drug. The average binding free energy of peramivir to neuraminidase calculated from structures taken between 20–40 ns from the H7N9-R292K trajectory is 2.4 ± 2.5 kcal mol^−1^ lower than that for the H7N9 trajectory. The difference in average binding free energy after the change in binding mode (50–70 ns) is much larger, is 9.7 ± 2.5 kcal mol^−1^.

## Discussion

Computational assay of drug binding to H11N9, H7N9 and H7N9-R292K neuraminidase shows that the differences in sequence between H11N9 and H7N9 have negligible impact on protein structure and drug binding, and that the neuraminidases from these strains are highly homologous. The structure developed in this study is thus likely to be a good model for H7N9 neuraminidase, providing a good foundation for further study of protein-drug interactions while a crystallographic structure remains unavailable. In addition, simulations show that the R292K substitution in H7N9-R292K neuraminidase disrupts binding of each of the currently approved neuraminidase antiviral compounds, suggesting weakened binding. This disruption is comparable to that observed experimentally between H11N9 and H11N9-R292K neuraminidase^20^,^21^. Waterswap calculations^23^ predict that this disruption, involving loss of specific interactions between the protein and the drugs, is associated with a reduction in binding free energy. This result is in line with the results of a recent experimental study^29^ that shows that the R292K mutation reduces the binding affinity of each of the drugs to H7N9 neuraminidase.

In addition to providing good models of the structure of H7N9 and H7N9-R292K neuraminidase, and a prediction of the reduction in binding affinity caused by the R292K mutation, this study demonstrates the recent advances in speed, accuracy and capability of modern molecular simulation techniques. While sequence data alone can only suggest that a strain of influenza may show weaker drug binding, computational structural and free energy studies can provide strong evidence. The necessary extensive molecular simulations can now test the effects of mutations rapidly: the required simulations can be completed within two weeks of a sequence being made publicly available with current computer resources (setup and running of the dynamics simulations here took one week, with a further week required for the WaterSwap calculations). Molecular dynamics simulations (using e.g. GPUs), coupled with binding calculations (using methods such as WaterSwap^23^) can now provide a rapid, predictive and practical assay on a useful timescale. This approach will be useful for neuraminidase and also other drug targets (e.g. enzymes) for which good quality experimental structures of the unmutated target exist and sequence data for emerging mutants typically becomes available before samples of the protein can be isolated for experimental assay or structural study. Molecular simulations of this type will complement experimental studies of emerging drug-resistance, and will assist in predicting possible deleterious mutations at the molecular level. Simulations can providing rapid predictions of the effects of changes in sequence, in particular predicting changes in structure and drug binding affinity, on a useful timescale. Molecular simulations can therefore now provide an assay to assist in predicting disruption of drug binding caused by the emergence of novel strains and new mutants of neuraminidase.

## Methods

The H7N9 neuraminidase sequences were downloaded from the GISAID EpiFlu™ database (www.gisaid.org) and aligned using Clustal Omega (www.clustal.org). H7N9 neuraminidase sequences A/Shanghai/1/2013 (EPI_ISL_138737), A/Shanghai/2/2013 (EPI_ISL_138738), A/Anhui/1/2013 (EPI_ISL_138739) and A/Hangzhou/1/2013 (EPI_ISL_138977) were aligned against the H11N9 sequence of A/Tern/Australia/G70C/1975, obtained from the X-ray structure 2QWK^20^ downloaded from the Protein Data Bank (PDB). This showed 95% sequence similarity in the head region (residues 83–468 using N2 numbering), with the Shanghai/2, Anhui/1 and Hangzhou/1 strains all identical in this region, and Shanghai/1 differing by a single amino acid (arginine to lysine at position R292, see table S1 in supporting information). X-ray structures of H11N9 A/Tern/Australia/G70C/1975 neuraminidase complexed with oseltamivir (2QWK^20^), zanamivir (1NNC^24^) and peramivir (1L7F^21^) were downloaded from the PDB and used as the starting structures of the H11N9 simulations. Copies of these were point mutated in-silico using the LEaP module of AMBER 12 to obtain the starting structures for the H7N9 and H7N9-R292K simulations. Mutated residues were built with starting geometries that matched the native residues. Each of the nine starting structures (H11N9, H7N9 and H7N9-R292K, each bound to oseltamivir, zanamivir and peramivir) were processed and simulated using the same protocol, which we have validated in a previous paper^25^. The coordinates of all crystallographic water and calcium ion molecules were retained. Hydrogen atoms were added independently for each system using LEaP, with an ionization state for each amino acid residue predicted by PROPKA^30^. Hydrogen atoms were added to oseltamivir, zanamivir and peramivir via a HF/6-31G* optimization using Gaussian 03. LEaP was used to solvate each system independently in a TIP3P^31^ water box of approximate volume 80 × 80 × 80 Å^3^, with chloride counter-ions added to neutralize the system. The FF03.r1 forcefield^32^ implemented in AMBER 12 was used to model neuraminidase. The atomic charges of the drugs were calculated based on the electrostatic potential from single point HF/6-31G* calculations using Gaussian 03, and fitted using the RESP module in AMBER 12. Parameters for the drugs were assigned from the generalized AMBER forcefield (GAFF)^33^ using Antechamber, with the parameters for oseltamivir and zanamivir tested in our previous validation study^25^. Energy minimization was performed to optimize the position of added hydrogen atoms and water molecules in each system using the pmemd.CUDA module in AMBER 12 (version 12.1, bugfix.9, released August 2012)^34^. The mixed single-precision/fixed precision (SPFP) version of pmemd.CUDA was employed. Particle-Mesh Ewald (PME) was used to account for long-range electrostatics, with a 10 Å real space cutoff, with the same cutoff used for the Lennard Jones potential. SHAKE was used to constrain bonds involving hydrogen. After minimization, molecular dynamics (MD) simulations were carried out also using pmemd.CUDA. A time step of 2 fs was used, with the simulation divided into thermalization, equilibration and production phases. In the thermalization step, the temperature was increased linearly to 310 K over a period of 100 ps using canonical (NVT) dynamics. A Langevin thermostat was used to maintain temperature, using a collision frequency of 5 ps^−1^. Next, the system was equilibrated for a further 100 ps of NVT dynamics, then a further 800 ps of isothermal-isobaric (NPT) dynamics, with a Langevin thermostat used to maintain a temperature of 310 K, and the isotropic pressure scaling algorithm implemented in pmemd.CUDA used to maintain the pressure at 1 bar, using a pressure relaxation time of 1 ps. Visual inspection of each system together with monitoring of RMSD confirmed that the starting structures closely matched the original crystal structures. Each system was then equilibrated for a further 20 ns at 310 K and 1 bar, before a further 50 ns of NPT dynamics was generated during the production phase. Thermalization, equilibration and production was carried out using a single M2090 nVidia Tesla GPU per simulation, with production dynamics of each ~60,000 atom system running at a rate of approximately 11 ns of sampling per day. The simulations were run in 1 ns blocks, with coordinates of all atoms saved every 10 ps. This generated approximately 3.7 GB of compressed data per simulation. In total, running the simulations required dedicated access to 9 M2090 GPUs for seven days, and generated about 33 GB of compressed data. WaterSwap^23^ calculations were performed using the WSRC module in Sire, with calculations using the same forcefield parameters and solvent model as the dynamics simulations. Six WaterSwap calculations were performed for each drug-neuraminidase complex, with the difference in mean average absolute binding free energy between the H7N9 and H7N9-R292K simulations reported (raw data supplied in table S2 in supporting information). Errors were estimated from the standard errors on these averages. WaterSwap^23^ calculations used starting structures taken from snapshots every 10 ns between 20–70 ns from each of the H7N9 and H7N9-R292K trajectories. Absolute binding free energies were calculated using replica-exchange thermodynamic integration^35^ over 16 λ windows (0.005, 0.071, 0.137, 0.203, 0.269, 0.335, 0.401, 0.467, 0.533, 0.599, 0.665, 0.731, 0.797, 0.863, 0.929, 0.995) over the WaterSwap reaction coordinate. 30 million Monte Carlo moves were performed for each window, with the free energy gradient averaged over the last 20 million steps. Simulations used the “Set A” soft-core parameters^23^, with a 15 Å coulomb and Lennard Jones non-bonded cutoff, the shifted-force cutoff^36^ used to account for long-range electrostatics, and a reflection sphere used to constrain sampling to within a 15 Å radius of the drug. Trajectories were visualized using VMD 1.9.1^37^. Visualisation and analysis was based on alignment of the trajectories against the protein backbone of the 2QWK structure, using the RMSD Trajectory Tool in VMD. Average occupations were generated using the VOLMAP tool in VMD, using a 0.5 Å grid, and calculating occupancy of all of the atoms from arginine/lysine 292 and oseltamivir, zanamivir or peramivir. Atoms were treated as hard spheres, with occupations calculated every 10 ps between 20–70 ns and averaged. All structural analysis was performed on frames taken every 10 ps from 20–70 ns from the trajectories. RMSDs were calculated using the RMSD tool in VMD, while RMSFs were calculated using the g_rmsf component of GROMACS. Interatomic distances were calculated using Wordom^38^, with distances between all equivalent donor-acceptor pairs evaluated, and a custom Python script used to select the minimum distance. These were processed and plotted using a custom Python script and Gnuplot, using a color scale capped from 2 Å (blue) to 8 Å (red). Distances greater than 8 Å were drawn using the same color as 8 Å, while distances less than 2 Å were drawn using the same color as 2 Å. The angle between the bulky group of oseltamivir and its central ring was calculated using VMD, using the atoms highlighted in figure S6 of supporting information.

## Author Contributions

C.J.W. and M.M. contributed equally to performing the experiments. B.L. provided technical support and conceptual advice. A.J.M., S.M.S., B.L., M.M. and C.J.W. contributed to the design and analysis of the experiments and preparation of the manuscript, with all five discussing the results and implications of the study and commenting on the manuscript at all stages.

## Supplementary Material

Supplementary Information Computational Assay of H7N9 Neuraminidase: Supplementary Information

## Figures and Tables

**Figure 1:**
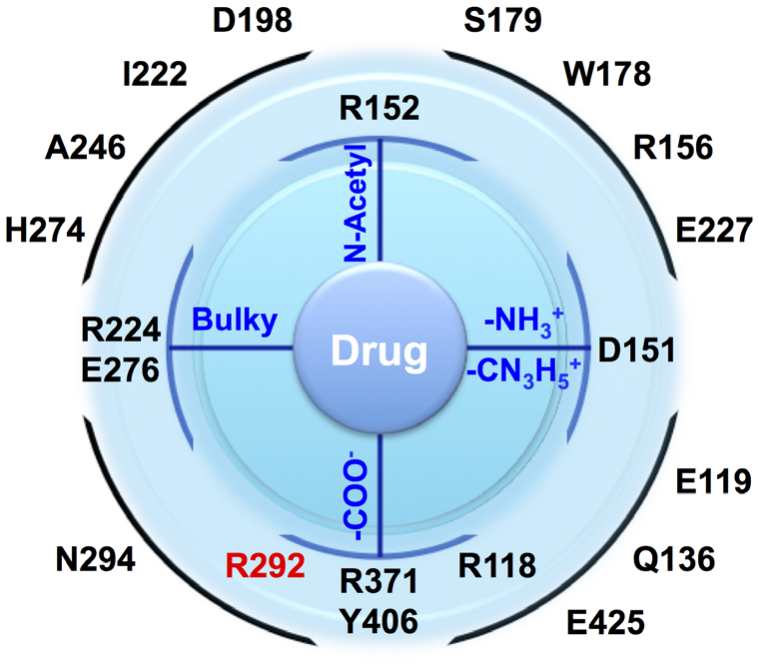
Schematic showing the approximate spatial location of the active site residues of neuraminidase in relation to the main functional groups of the antiviral compounds (see figure S1 for the chemical structure of the drugs). Residues that form direct interactions with the drugs are drawn inside the outer circle. The R292 residue is shown in red. Residues are numbered using N2 numbering.

**Figure 2:**
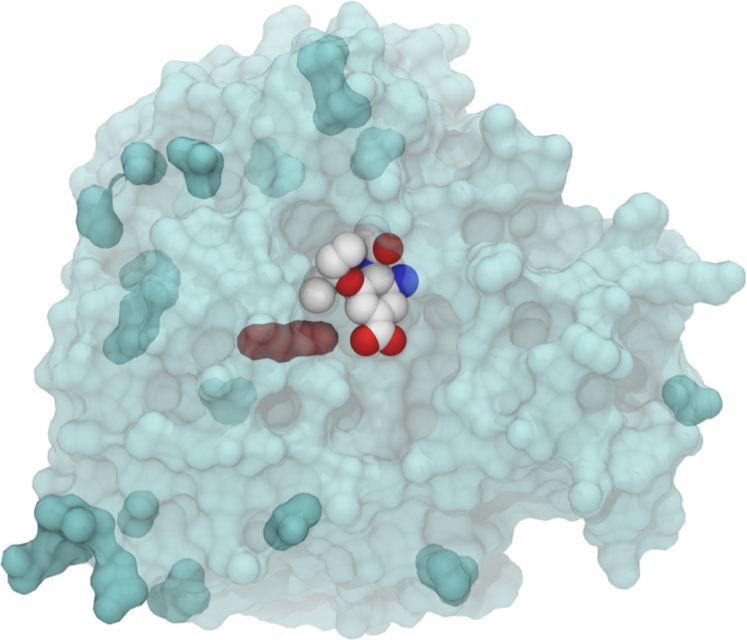
Oseltamivir bound to H7N9-R292K neuraminidase. This initial computational model was built by point mutating individual residues of H11N9 neuraminidase bound to oseltamivir (PDB structure 2QWK^20^) to match the sequence of H7N9-R292K. Residues that differ from the reference H11N9 neuraminidase are shown in dark cyan, with the R292K lysine residue highlighted in red. This structure provides the starting point for the H7N9-R292K oseltamivir-bound simulation (see Methods section for more details).
