# Supplementary material for: Computational Assay of H7N9 Influenza Neuraminidase Reveals R292K Mutation Reduces Drug Binding Affinity
Source: Sci Rep. 2013 Dec 20;3:3561. doi: 10.1038/srep03561 (PMC3868970; doi:10.1038/srep03561)
Supplement: Supplementary Information — Computational Assay of H7N9 Neuraminidase: Supplementary Information [file srep03561-s1.pdf]

SUPPLEMENTARY INFORMATION

Computational Assay of H7N9 Influenza Neuraminidase Reveals R292K Mutation Reduces Drug Binding Affinity

Christopher Woods[1], Maturos Malaisree[1], Ben Long[2], Simon McIntosh-Smith[2], Adrian Mulholland[1]

School of Chemistry[1] and Department of Computer Science[2], University of Bristol, Bristol, UK

Residue Number	H11N9	H7N9	H7N9-R292K
83	D	N	N
111	D	S	S
188	T	S	S
205	I	V	V
213	T	A	A
251	E	D	D
267	P	S	S
269	A	T	T
285	A	T	T
286	E	G	G
292	R	R	K
304	R	Q	Q
332	T	N	N
333	V	I	I
358	V	A	A
368	I	T	T
387	K	R	R
391	T	I	I
401	T	A	A
416	E	D	D
457	D	N	N

Table S1: Differences in sequence in the head region of neuraminidase between A/Tern/Australia/G70C/1975 (H11N9) and A/Shanghai/2/2013 (H7N9) and A/Shanghai/1/2013 (H7N9-R292K), matching with residues numbers from structure 2QWK.

Drug	Time / ns	ΔG_{H7N9} / kcal mol^{-1}	$\Delta G_{H7N9\text{-}RK}$ / kcal mol^{-1}
**Oseltamivir**	20	-24.5	-25.6
	30	-30.6	-22.2
	40	-24.9	-19.9
	50	-25.1	-21.7
	60	-20.2	-18.7
	70	-23.4	-22.5
	**Mean Average**	**-24.8**	**-21.8**
	**Standard Error**	**1.4**	**1.0**
**Zanamivir**	20	-33.9	-29.9
	30	-38.3	-28.6
	40	-16.9	-25.9
	50	-27.1	-32.7
	60	-28.5	-11.3
	70	-35.5	-25.0
	**Mean Average**	**-30.0**	**-25.6**
	**Standard Error**	**3.1**	**3.1**
**Peramivir**	20	-28.0	-25.0
	30	-38.0	-24.5
	40	-30.9	-34.0
	50	-31.7	-25.2
	60	-23.1	-21.2
	70	-29.7	-15.1
	**Mean Average**	**-30.2**	-24.2
	**Standard Error**	**2.0**	2.5
	**Mean Average 20-40 ns**		**-27.8**
	**Standard Error 20-40 ns**		**3.1**
	**Mean Average 50-70 ns**		**-20.5**
	**Standard Error 50-70 ns**		**2.9**

Table S2: Absolute binding free energies calculated via WaterSwap for each drug to H7N9 and H7N9-R292K neuraminidase. Free energies are calculated starting from snapshots taken every 10 ns between 20-70 ns from the corresponding molecular dynamics trajectory. Raw absolute binding free energies are presented, with no corrections for changes in accessible volume or ionic strength as the drug is swapped from bulk water to the protein binding site. This means that the WaterSwap free energies overestimate the true binding free energy by a value that is constant for each drug, thereby allowing differences in binding for each drug to each mutant to be calculated. The average binding free energy for peramivir to H7N9-R292K neuraminidase is reported from 20-40 ns and 50-70 ns as the binding mode of the drug changed after approximately 45 ns of dynamics.

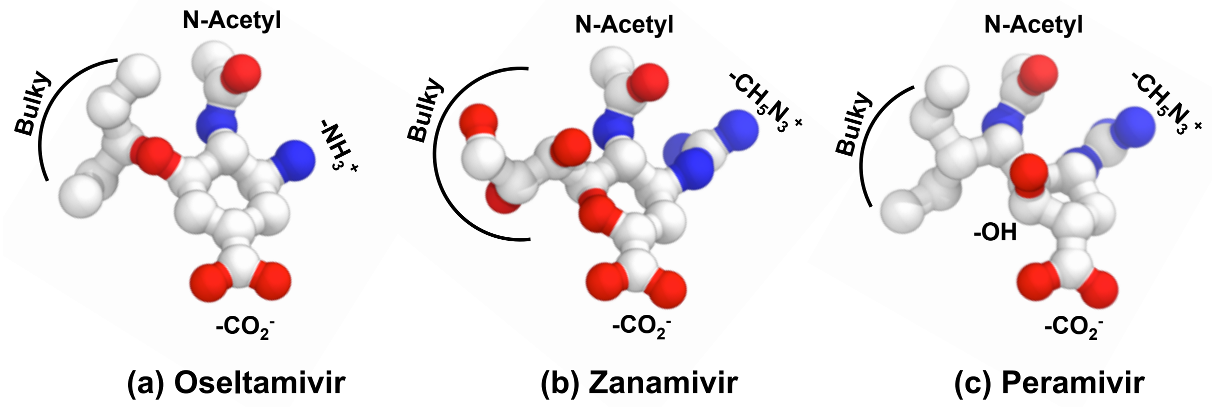

Figure S1: Structures of (a) oseltamivir, (b) zanamivir and (c) peramivir , highlighting the locations of the bulky, N-acetyl, carboxylate ($-CO_2^-$), ammonium ($-NH_3^+$), guanidinium ($-CH_5N_3^+$) and hydroxyl (-OH) group substituents. Only heavy (non-hydrogen) atoms are shown.

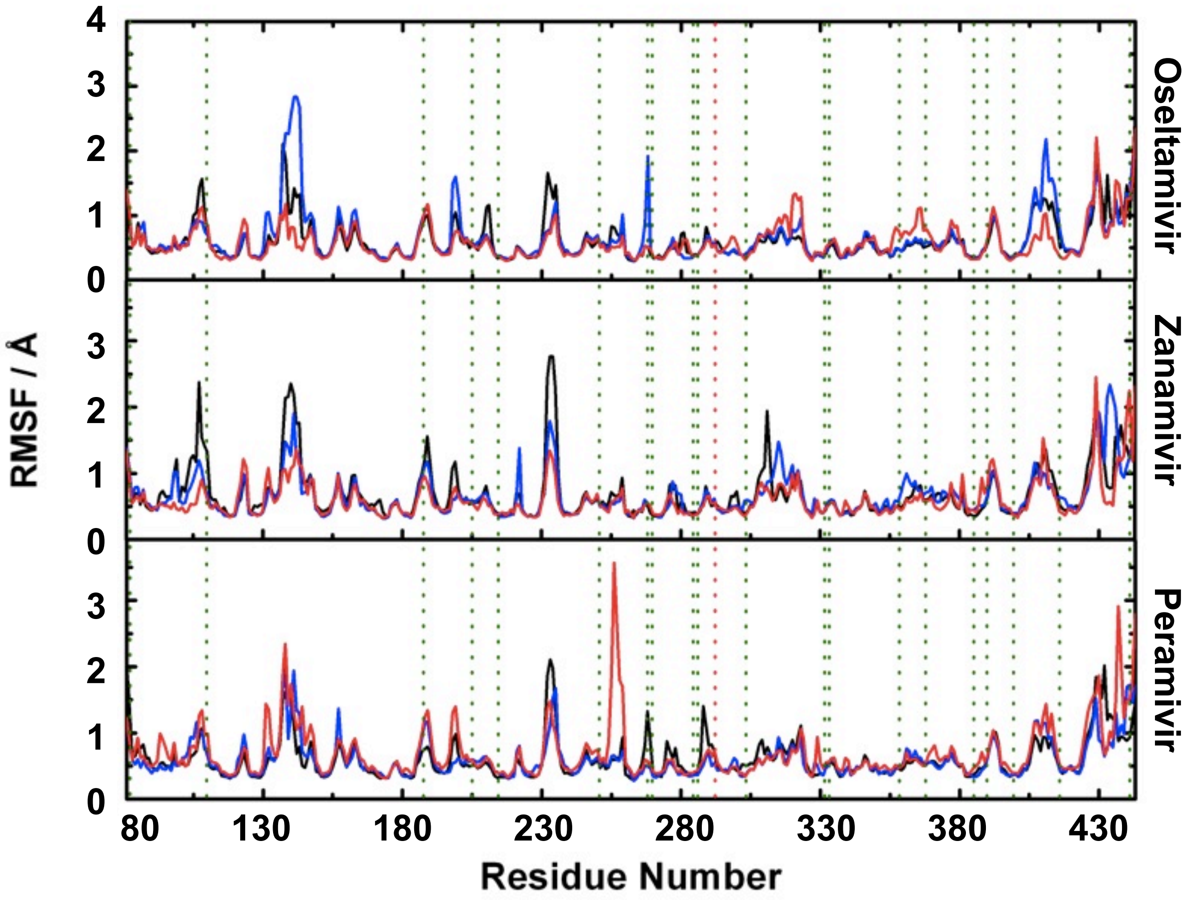

Figure S2: Root mean square fluctuations (RMSF) of all of the simulated residues of neuraminidase from all the H11N9 (black lines), H7N9 (blue lines) and H7N9-R292K (red lines) trajectories. The RMSF was calculated every 10 ps from 20-70 ns. The residues that are different between the H11N9, H7N9 and H7N9-R292K structures are highlighted using green dotted lines, while R292K is highlighted with a red dotted line. The RMSFs from all trajectories are very similar, with the only major difference being residues Y255, Y256 and F257 in the H7N9-R292K peramivir simulation. Visual inspection of these residues confirmed that they were far from the active site and caused little disruption to the structure of the protein.

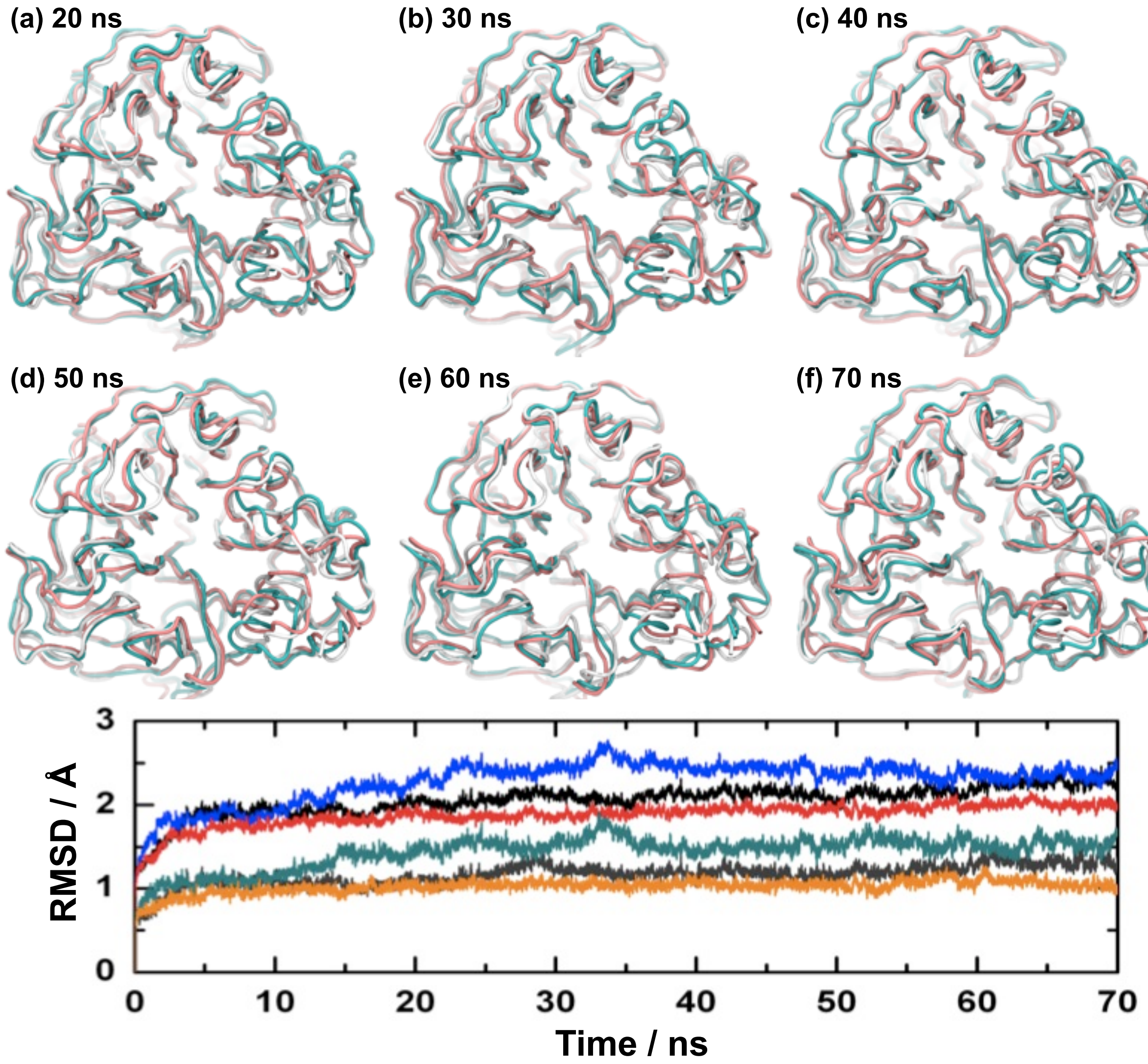

Figure S3: The conformation of the backbone of neuraminidase taken every 10 ns between 20-70 ns from the oseltamivir bound trajectories. The conformation of the H11N9 (white), H7N9 (cyan) and H7N9-R292K (pink) is shown. The graph below shows the root mean square deviation (RMSD) of all of the protein atoms (dark colors) and all of the protein backbone atoms (light colors), with H11N9 in black/grey, H7N9 in blue/cyan and H7N9-R292K in red/orange. RMSDs are calculated relative to the original (post-minimization) starting structure of the simulation.

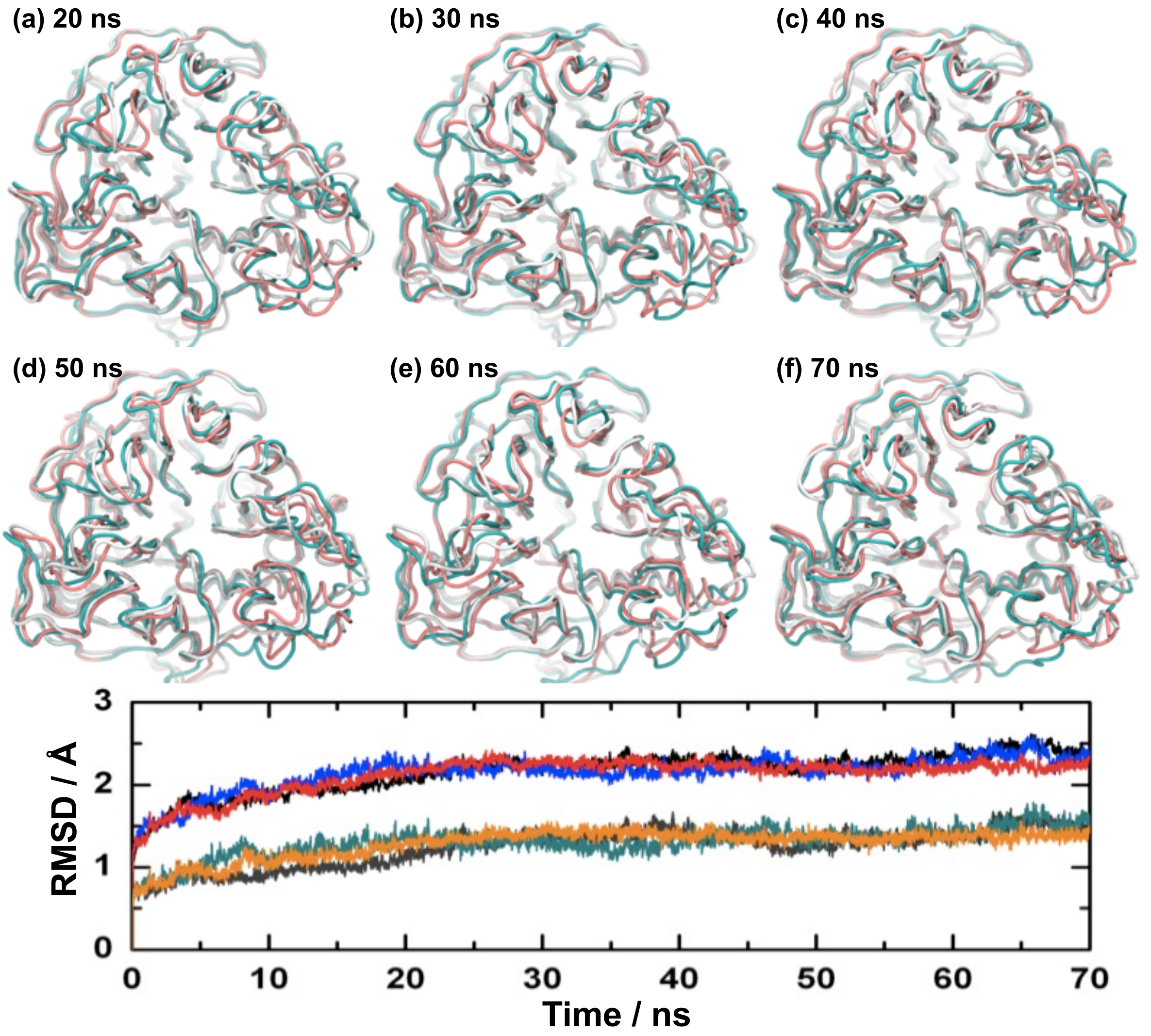

Figure S4: The conformation of the backbone of neuraminidase taken every 10 ns between 20-70 ns from the zanamivir bound trajectories. The conformation of the H11N9 (white), H7N9 (cyan) and H7N9-R292K (pink) is shown. The graph below shows the root mean square deviation (RMSD) of all of the protein atoms (dark colors) and all of the protein backbone atoms (light colors), with H11N9 in black/grey, H7N9 in blue/cyan and H7N9-R292K in red/orange. RMSDs are calculated relative to the original (post-minimization) starting structure of the simulation.

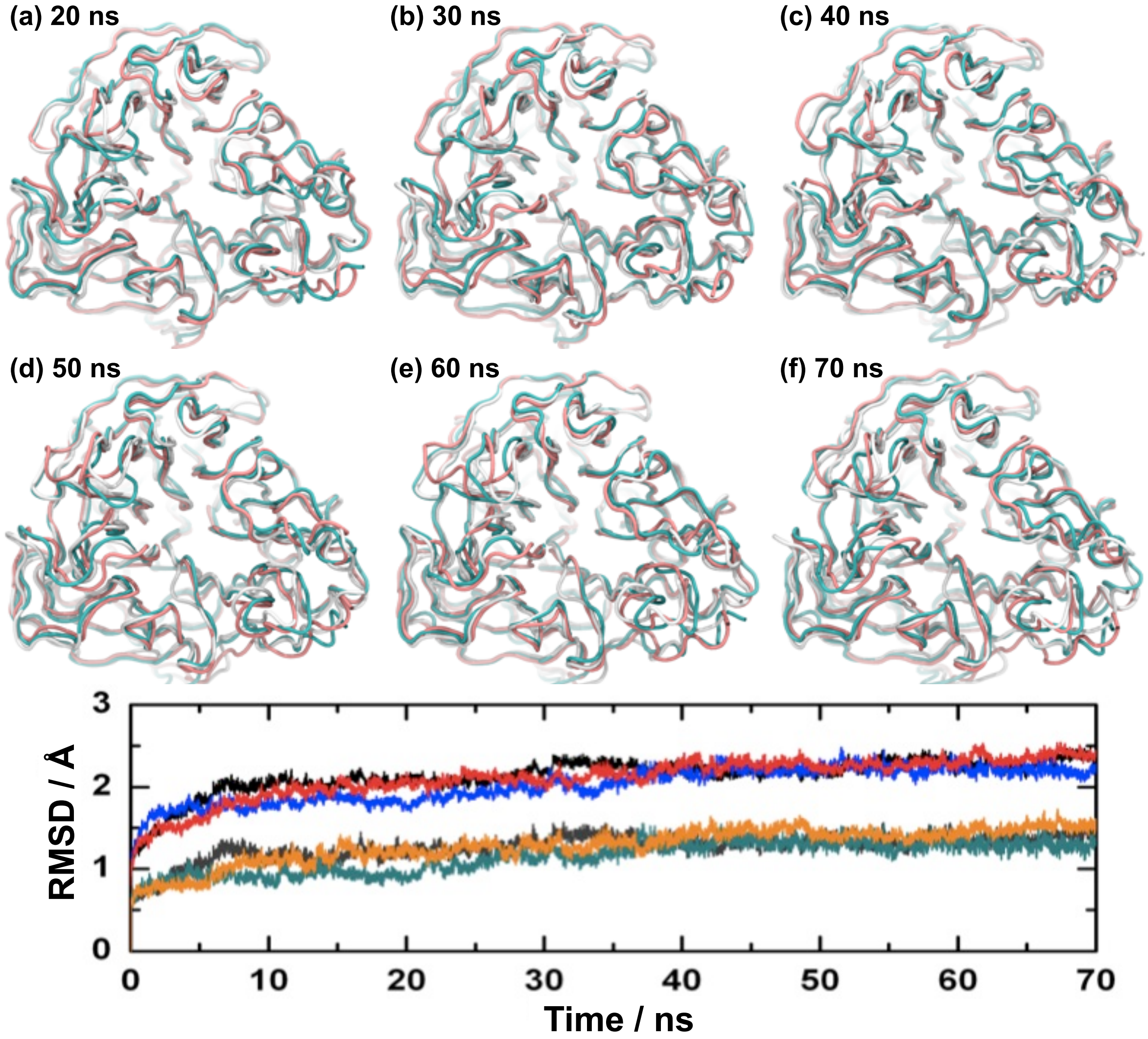

Figure S5: The conformation of the backbone of neuraminidase taken every 10 ns between 20-70 ns from the peramivir bound trajectories. The conformation of the H11N9 (white), H7N9 (cyan) and H7N9-R292K (pink) is shown. The graph below shows the root mean square deviation (RMSD) of all of the protein atoms (dark colors) and all of the protein backbone atoms (light colors), with H11N9 in black/grey, H7N9 in blue/cyan and H7N9-R292K in red/orange. RMSDs are calculated relative to the original (post-minimization) starting structure of the simulation.

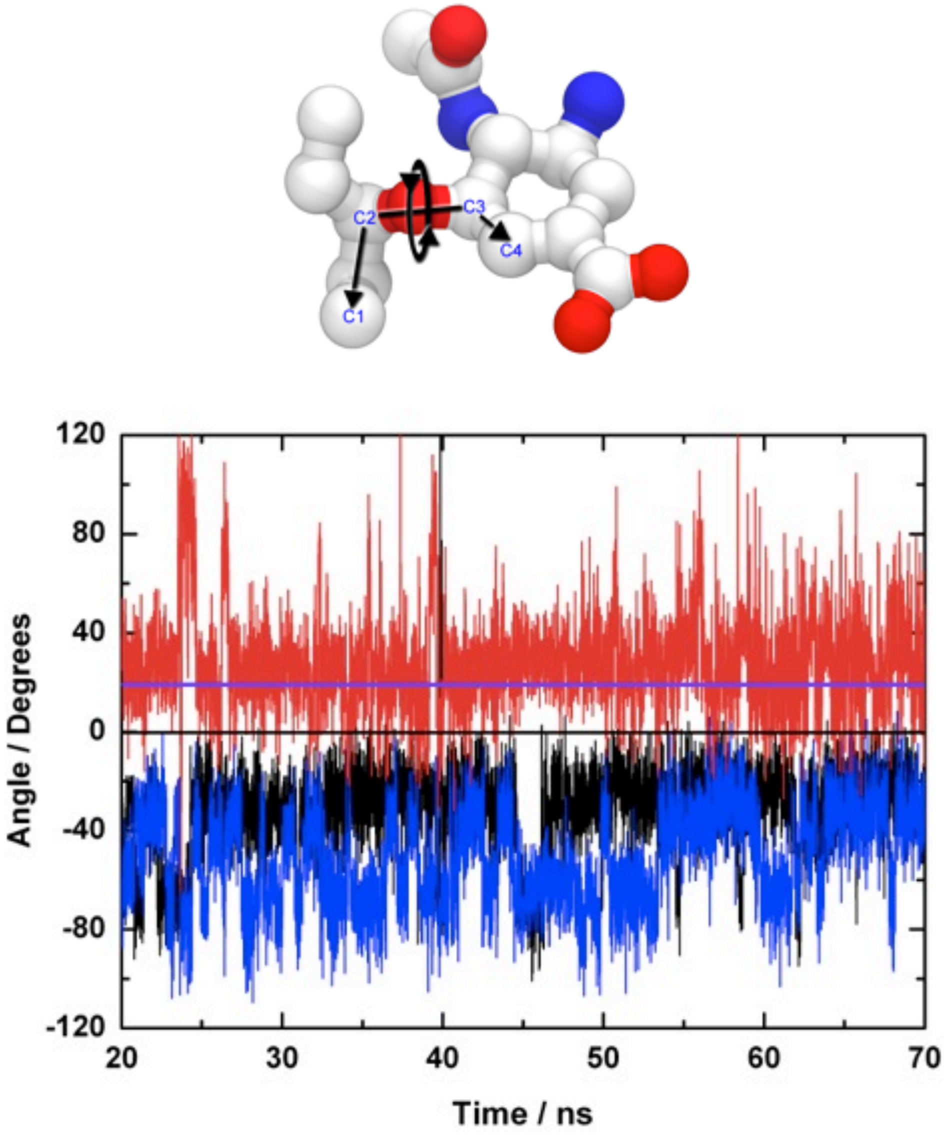

Figure S6: Angle between the bulky group and the ring of oseltamivir as calculated every 10 ps between 20-70 ns from the H11N9 (black), H7N9 (blue) and H7N9-R292K (red) trajectories. For comparison, the equivalent angle calculated for the X-ray structure of H11N9-R292K (19.1°) is shown as a straight line (purple). This is calculated as the angle between the vectors from the C1-C2 and C3-C4 atoms highlighted in the picture of oseltamivir above.

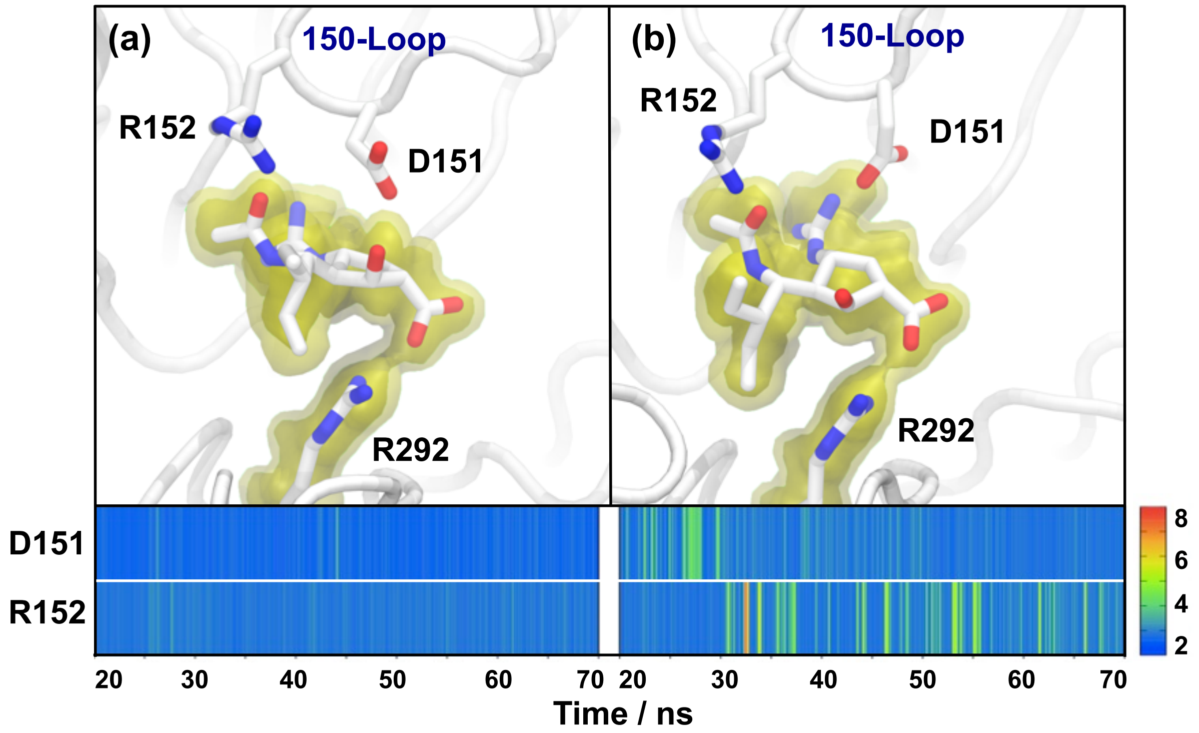

Figure S7: Peramivir complexed with (a) H11N9 and (b) H7N9 neuraminidase. A representative snapshot from simulation is displayed, together with the average location of peramivir and lysine shown using isosurfaces, with the volume of space occupied during 90% (solid yellow) and 60% (transparent yellow) of the trajectory displayed. The shortest distances between the hydrogen bond donors and acceptors of peramivir (guanidinium and hydroxyl groups) to the D151 and R152 residues on the 150-loop was calculated every 10 ps between 20-70 ns and is also plotted. Distances are shown on a color-scale from 2-8 Å, with blue-green values indicating the presence of a hydrogen bond.
